# Artificial intelligence for X-ray scaphoid fracture detection: a systematic review and diagnostic test accuracy meta-analysis

**DOI:** 10.1007/s00330-023-10473-x

**Published:** 2023-12-15

**Authors:** Matan Kraus, Roi Anteby, Eli Konen, Iris Eshed, Eyal Klang

**Affiliations:** 1 Department of Diagnostic Imaging, Sheba Medical Center, 2 Sheba Road, 5262000 Ramat Gan, Israel; 2 Sackler School of Medicine, Tel Aviv University, Tel Aviv, Israel; 3 Department of General Surgery, Sheba Medical Center, 2 Sheba Road, 5262000 Ramat Gan, Israel

**Keywords:** Scaphoid bone, Wrist fractures, Machine learning, Deep learning

## Abstract

**Objectives:**

Scaphoid fractures are usually diagnosed using X-rays, a low-sensitivity modality. Artificial intelligence (AI) using Convolutional Neural Networks (CNNs) has been explored for diagnosing scaphoid fractures in X-rays. The aim of this systematic review and meta-analysis is to evaluate the use of AI for detecting scaphoid fractures on X-rays and analyze its accuracy and usefulness.

**Materials and methods:**

This study followed the guidelines of Preferred Reporting Items for Systematic Reviews and Meta-analyses (PRISMA) and PRISMA-Diagnostic Test Accuracy. A literature search was conducted in the PubMed database for original articles published until July 2023. The risk of bias and applicability were evaluated using the QUADAS-2 tool. A bivariate diagnostic random-effects meta-analysis was conducted, and the results were analyzed using the Summary Receiver Operating Characteristic (SROC) curve.

**Results:**

Ten studies met the inclusion criteria and were all retrospective. The AI’s diagnostic performance for detecting scaphoid fractures ranged from AUC 0.77 to 0.96. Seven studies were included in the meta-analysis, with a total of 3373 images. The meta-analysis pooled sensitivity and specificity were 0.80 and 0.89, respectively. The meta-analysis overall AUC was 0.88. The QUADAS-2 tool found high risk of bias and concerns about applicability in 9 out of 10 studies.

**Conclusions:**

The current results of AI’s diagnostic performance for detecting scaphoid fractures in X-rays show promise. The results show high overall sensitivity and specificity and a high SROC result. Further research is needed to compare AI’s diagnostic performance to human diagnostic performance in a clinical setting.

**Clinical relevance statement:**

Scaphoid fractures are prone to be missed secondary to assessment with a low sensitivity modality and a high occult fracture rate. AI systems can be beneficial for clinicians and radiologists to facilitate early diagnosis, and avoid missed injuries.

**Key Points:**

*• Scaphoid fractures are common and some can be easily missed in X-rays.*

*• Artificial intelligence (AI) systems demonstrate high diagnostic performance for the diagnosis of scaphoid fractures in X-rays.*

*• AI systems can be beneficial in diagnosing both obvious and occult scaphoid fractures.*

**Supplementary Information:**

The online version contains supplementary material available at 10.1007/s00330-023-10473-x.

## Introduction

Scaphoid fractures are the most common type of carpal bone fracture, with an incidence of 82–89% [1]. Early detection and immobilization are crucial for the successful management of scaphoid fractures [2–4] since neglected untreated fracture of the scaphoid may lead to complications such as avascular necrosis, carpal instability, and early osteoarthritis [4–8].

X-rays are commonly used to identify scaphoid fractures due to their accessibility and cost-effectiveness. However, the sensitivity for diagnosing scaphoid fracture on wrist radiographs is relatively low (66–81%) with a likelihood of up to 50% of occult scaphoid fractures [9–13]. Radiographic wrist interpretation of scaphoid fracture poses a challenge and is a frequent cause of delayed or incorrect diagnosis in emergency departments. Radiographs are often evaluated by non-specialized physicians or junior orthopedic residents potentially affecting accuracy and leading to missed fractures [14]. Thus, possibly due to the presence of less experienced personnel or fatigue, there is a higher incidence of misdiagnosis during evening and overnight hours, on top of the relatively constant shortage of emergency radiologists available to handle the workload [15].

While advanced imaging modalities such as CT or MRI are more sensitive than radiographs for the detection of occult scaphoid fractures, there are inherent disadvantages in these modalities such as increased radiation exposure by CT and high medical cost by MRI [2, 16, 17].

Therefore, there is both clinical and economic advantage in improving the sensitivity and detection rate of scaphoid fractures on plain radiographs. Convolutional Neural Networks (CNNs) are prominent artificial intelligence (AI) deep learning algorithms for image analysis [18, 19]. CNNs are specifically designed to handle image data by exploiting repeating patterns. They have been used for various medical image processing, such as radiology images, skin lesions, retinal scans, endoscopic images, and histopathologic specimens [19–29].

AI algorithms have proven to be effective in identifying various acute pathologies that are frequently encountered in emergency departments, for example, pulmonary embolism, intra-abdominal free gas, intra-cranial hemorrhage, and femoral neck fractures [15]. Indeed, AI methods have been explored for improving sensitivity in the detection of scaphoid fractures on wrist radiographs.

The aim of this systematic review and meta-analysis is to comprehensively evaluate the existing data on the use of AI for detecting scaphoid fractures on wrist radiographs. The review provides a comprehensive analysis of the usefulness and accuracy of current AI systems in this field, and explores its potential applications in the future.

## Materials and methods

The systematic review followed the guidelines of Preferred Reporting Items for Systematic Reviews and Meta-analyses (PRISMA) and PRISMA-Diagnostic Test Accuracy [30, 31]. A literature search was conducted in the PubMed database for relevant studies published until July 2023, using search terms: (“Scaphoid Bone”[Mesh] or “Wrist”[Mesh] or “scaphoid” or “scaphoid fracture” or “Wrist Fractures”[Mesh]) and (“deep learning”[Mesh] or “artificial intelligence”[Mesh] or “convolutional neural network” or “CNN” or “Neural Networks, Computer”[Mesh]). The bibliographies of the included studies were also searched for additional relevant studies.

Inclusion criteria for studies were (1) use of AI methods for scaphoid fracture diagnosis on wrist radiographs, (2) reporting of statistical analysis of area under the ROC (receiver operating characteristic) and/or accuracy, (3) publication in English, and (4) original articles.

Data from all included studies was collected using a standardized data extraction sheet, including publication year, journal name and affiliation, study design, study period, number and views of wrist radiographs, number of patients and images, fracture type (visible or occult), standard of reference for diagnosis, number of pipeline steps, AI name, CNN architecture, ROI labeling, data input proportion, diagnostic accuracy/AUC, sensitivity, specificity, and true/false positive/negative. The Quality Assessment of Diagnostic Accuracy Studies (QUADAS-2) was used to assess bias and applicability [32].

### Statistical analysis

The results of the systematic review and meta-analysis on AI for the detection of scaphoid fractures in wrist radiographs were analyzed based on the contingency table of true positives, false negatives, true negatives, and false positives constructed for each study.

A bivariate diagnostic random-effects meta-analysis was conducted and the findings were assessed with the Summary Receiver Operating Characteristic (SROC) curve [33]. The SROC curve considered the sensitivity and specificity values for a given test, taking into account the various cut-off points in each independent study. The area under the curve (AUC) was calculated for the fitted SROC. AUC is an overall summary of diagnostic accuracy, commonly used when reporting the performance of AI classification models. The ROC curve is plotted with the true positive rate (sensitivity) against the false positive rate (1-specificity). The higher the AUC, the better the model’s performance is at distinguishing between scaphoid fracture and non-fracture plain radiographs. AUC results are considered excellent for values between 0.9 and 1, good for AUC values between 0.8 and 0.9, and fair for AUC values between 0.8 and 0.7. An AUC less than 0.7 is considered poor. Accuracy is defined as the number of correct predictions divided by the total prediction number (true positives + true negatives/total dataset). The across-study heterogeneity was assessed using the *I*^2^ statistic. The analyses were performed using the R 4.0.5 software and the “mada” and “meta” packages.

## Results

### Included studies and dataset sizes

**Fig. 1:**
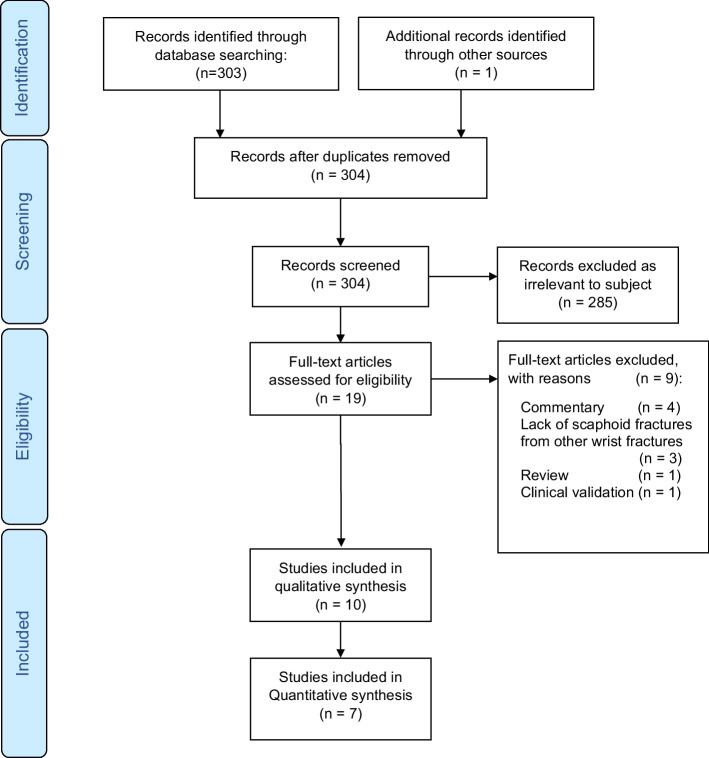
Flow diagram of the search and inclusion process. The study was reported according to the Preferred Reporting Items for Systematic Reviews and Meta-analyses guidelines

The bibliographic search retrieved 303 studies, of which nine studies met the inclusion criteria and one more was retrieved by manual searching [2, 16, 34–41]. A flow diagram of the study selection process is presented in Fig. 1, and the characteristics of the included studies are summarized in Table 1. The studies were all retrospective and published between 2020 and 2023.

**Table 1:** Included study characteristics

Study	Publication year	Journal	Journal affiliation	Study design
Langerhuizen et al [34]	2020	Clinical Orthopaedics and Related Research	Clinical	Retrospective
Ozkaya et al [16]	2020	European Journal of Trauma and Emergency Surgery	Clinical	Retrospective
Yoon et al [35]	2021	JAMA Open network	Clinical	Retrospective
Hendrix et al [36]	2021	Radiology: Artificial Intelligence	Clinical	Retrospective
Tung et al [37]	2021	Applied Sciences	Bio-computational	Retrospective
Yang et al [38]	2022	Diagnostics	Bio-computational	Retrospective
Li et al [2]	2022	Journal of Hand Surgery	Clinical	Retrospective
Hendrix et al [39]	2023	European Radiology	Clinical	Retrospective
Cohen et al [40]	2023	European Radiology	Clinical	Retrospective
Lee et al [41]	2023	Diagnostics	Bio-computational	Retrospective

The ten included studies ranged in sample size, with the number of images used varying from 356 to 11,838. Table S1 (Supplementary Material) provides a summary of the full numerical data of the studies, including the reference standard (“ground truth”) for diagnosis.

### Brief summary of the included studies

Langerhuizen et al [34] developed a single-step CNN model that consisted of only the scaphoid fracture detection step; scaphoid segmentation was done manually. The model showed fair diagnostic accuracy, with an AUC of 0.77, and diagnostic performance was compared to five orthopedic surgeons. The clinicians had better specificity, but the CNN performed similarly in terms of accuracy and sensitivity. However, the algorithm struggled with identifying obvious fractures and had some false positive suggestions.

Ozkaya et al [16] also developed a single-step fracture detection CNN, while scaphoid segmentation was done manually. The CNN’s diagnostic performance was compared with an emergency department physician and two hand surgeons. The CNN had a good AUC of 0.84. The CNN’s performance was comparable to that of a less experienced orthopedic specialist and outperformed the emergency department physician.

Yoon et al [35] developed a 3-step CNN model. Firstly, the scaphoids were localized by a segmentation CNN. Afterwards, two CNNs were used consecutively, one to detect scaphoid fractures, followed by another CNN which examined the negative cases of the previous CNN, in order to detect the occult fractures. The fracture detection CNN achieved excellent diagnostic performance with an AUC of 0.955. The “occult fracture detection” CNN, had a good AUC of 0.81. The full model successfully identified 90.9% of occult fractures.

Hendrix et al [36] developed a 2-step CNN model and compared its diagnostic performance to that of eleven radiologists with various experiences. The first CNN localized the scaphoids and automatically passed the region of interest to the fracture detection CNN. The CNN had a good AUC of 0.87 and demonstrated a performance level comparable to that of the radiologists.

Tung et al [37] developed a 2-step CNN model: scaphoid segmentation CNN followed by scaphoid fracture detection CNN. Ten different CNNs for fracture detection were used and compared, with an AUC ranging from 0.86 to 0.95.

Yang et al [38] developed a 2-step CNN model: scaphoid segmentation CNN and scaphoid fracture detection CNN, which achieved an excellent diagnostic performance with an AUC of 0.917.

Li et al [2] developed a 2-step CNN model: scaphoid segmentation and scaphoid fracture detection; and compared its diagnostic performance to that of four hand surgeons. The fracture detection CNN achieved an excellent diagnostic performance with an AUC of 0.92. They concluded that CNN’s ability was comparable to the majority decision of the surgeons, and has the potential to achieve expert-level performance.

Hendrix et al [39] developed a 2-step CNN model: scaphoid segmentation and laterality classification CNN, and scaphoid fracture detection CNN, and assessed the diagnostic performance of the AI system. They also conducted an observer study in order to clinically validate the AI system performance, and compared the diagnostic performance of five musculoskeletal-expert radiologists with and without AI assistance. The CNN had a good AUC of 0.88, and it was comparable to the average performance (AUC 0.87) of the five radiologists. The AI assistance improved the agreement between radiologists in 5/10 pairs, and it reduced reading time for 4/10 radiologists. However, the algorithm did not improve the diagnostic performance of the majority of radiologists.

Cohen et al [40] used a commercially available AI system and assessed its diagnostic performance in detecting different wrist fractures, including scaphoid fractures. They also conducted an observer study in order to clinically validate the AI system, and compared the diagnostic performance of initial radiology reports (IRR) made by 41 radiologists with various experiences, which are not specialized in musculoskeletal imaging. Afterwards, the IRRs the AI results alone, and the combination of the two were compared. IRR + AI observation was considered positive when it was detected by either the AI or the IRR, regardless of the other’s group result. The sensitivity for scaphoid fracture detection was similar for both AI (84%) and IRR (80%). Analysis of the full cohort, meaning all fracture locations, showed that the combination of IRR + AI had greater sensitivity compared to either AI alone or IRR alone.

Lee et al [41] developed an AI model to detect three common types of wrist fractures: distal radius, ulnar styloid process, and scaphoid fractures. The model consisted of two CNNs: scaphoid segmentation and scaphoid fracture detection, which operated simultaneously and were integrated into a final assessment. Two novice radiologists also diagnosed the fracture sites, both with and without the assistance of the AI model. The diagnostic performance of the AI model was evaluated and compared to the novice radiologists. The AI model had a good AUC of 0.808 for scaphoid fracture detection. When novice radiologists were assisted by the AI model, the AUC for detecting scaphoid fractures significantly increased from 0.75 to 0.85 and from 0.71 to 0.80.

### Radiograph characteristics

Two studies [34, 39] used a full wrist series of 4 projections (either anterior–posterior (AP), posterior-anterior (PA), lateral and oblique or AP/PA, ulnar-deviated AP/PA, lateral and oblique). Two studies [2, 35] used 2 projections (PA or scaphoid view), one study [41] used 3 projections of wrist radiographs (AP, lateral, oblique), one study [36] used several hand, wrist, and scaphoid projections, one study [37] used 2 projections (AP and lateral), and one study [16] used 1 projection (AP). Two studies [38, 40] did not specify which projections were used. Exclusion criteria were specified in seven studies [2, 34–36, 39–41] and included poor radiographic quality, old fractures, immobilization devices, hardware, chronic hand disorders, arthritis, and tumors. The other three studies [16, 37, 38] did not specify any exclusion criteria.

### CNN pipeline structure and AI characteristics

One study [40] used commercially available software, while the other nine studies [2, 16, 34–39, 41] developed CNNs using radiographs for AI training and testing.

Data on the AI characteristics is shown in Table S2 (Supplementary Material). Nine different CNN architecture types were used in the AI models that were developed, along with various augmentation techniques. Eight studies [2, 16, 34–36, 38, 39, 41] utilized a single CNN architecture, which differed in each study. One study [37] compared 10 different CNN architecture types.

The most common CNN models used among the studies were VGG16 [34, 37], Resnet-50 [16, 37], DenseNet-121 [36, 37], ResNet-152 [37, 38], and Inception-V3 [37, 39], with two each. Three studies [2, 35, 36] used the Grad-Cam function to create a heatmap based on the input image and highlight the fracture area. Another heatmap was used in one study [41], and a bounding box to highlight the “zone-of-interest” was integral to the commercially available AI system that was used in a single study [40].

Two studies [34, 36] used a 1-step pipeline model with a CNN solely for fracture detection. Scaphoid segmentation was done manually, by cropping and resizing the scaphoid to fit in a rectangular ROI, which then served as input to the fracture detection CNN.

Six studies [2, 36–39, 41] used a 2-step pipeline model: a scaphoid segmentation CNN followed by a fracture detection CNN.

**Fig. 2:**
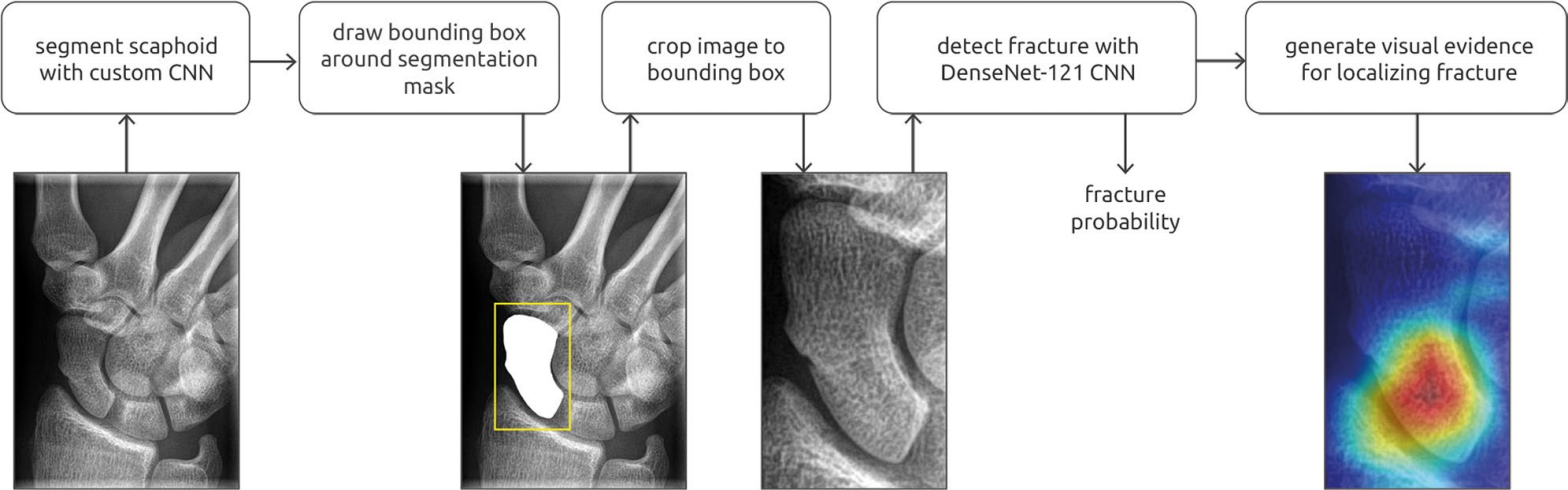
Example of scaphoid fracture detection pipeline, which consists of segmentation and detection of Convolutional Neural Networks (CNN). A class activation map is calculated and visualized as a heatmap for fracture localization. Reproduced with permission: Fig. 1, Hendrix N, Scholten E, Vernhout B, et al. Development and Validation of a Convolutional Neural Network for Automated Detection of Scaphoid Fractures on Conventional Radiographs. Radiology: Artificial Intelligence: Published Online April 28, 2021. https://doi.org/10.1148/ryai.2021200260. © Radiological Society of North America

An example of such CNN is illustrated in Fig. 2 [36].

One study [35] used a 3-step pipeline model, with a scaphoid segmentation CNN followed by a fracture detection CNN. The negative results from the first two steps were re-evaluated in a third CNN, designed to diagnose occult fractures missed in previous pipeline steps. The entire pipeline was then tested separately on new images. One study used a commercial system, in which scaphoid segmentation and fracture detection were integral [40].

### AI performance for scaphoid fracture detection

**Fig. 3:**
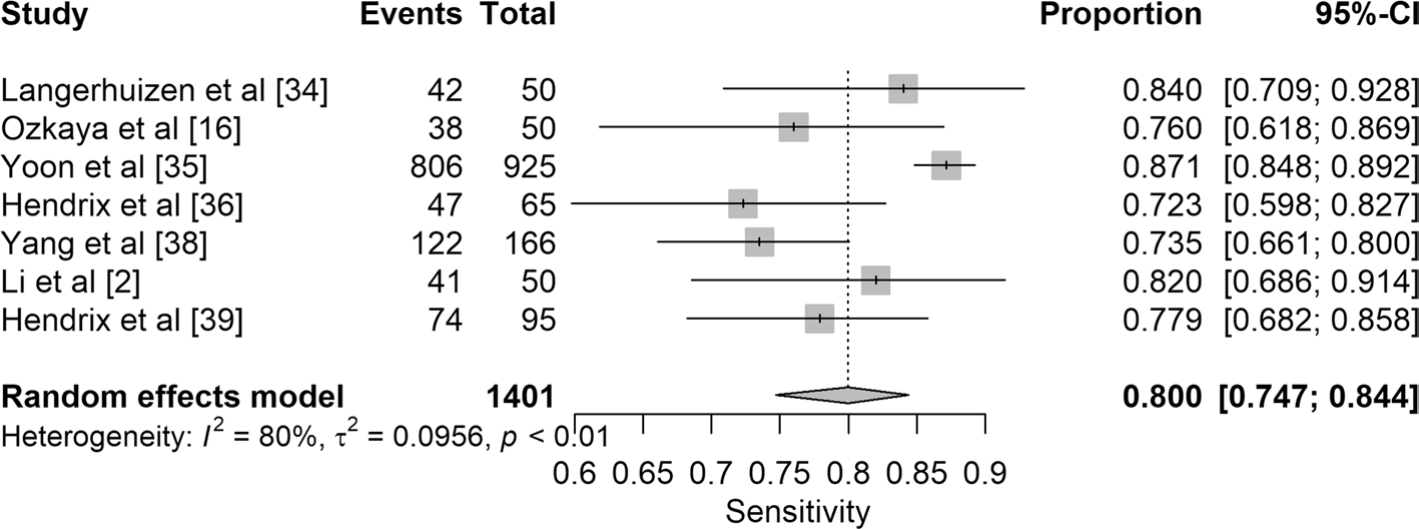
Forest plot of the pooled sensitivity of studies reporting on deep learning detection of scaphoid fractures

**Fig. 4:**
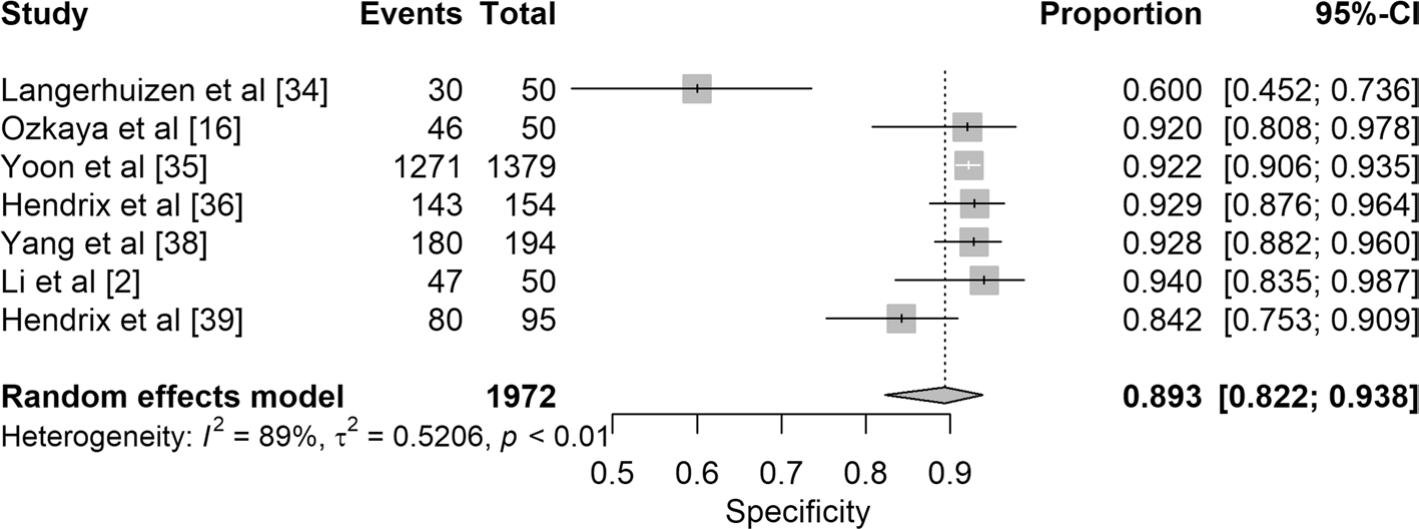
Forest plot of the pooled specificity of studies reporting on deep learning detection of scaphoid fractures

Data on AI compared to human performance is shown in Table S3 (Supplementary Material). The AI performance of scaphoid fracture detection varied from AUC 0.77 to 0.96. Accuracy of fracture detection ranged from 72.0 to 90.3%. Sensitivity and specificity for each CNN are shown in forest plots (Figs. 3 and 4).

Seven studies reported deep learning algorithm performance, with a combined total of 3373 images. The pooled sensitivity and specificity were 0.80 (95% CI 0.75–0.84) and 0.89 (95% CI 0.82–0.94) respectively **(**Figs. 3 and 4).

Tung et al [37] compared 10 different CNNs applied to the same dataset and found AUCs ranging from 0.86 to 0.95. The other eight studies [2, 16, 34–36, 38, 39, 41] also reported high AUC, from 0.77 to 0.96.

The AUCs were fair for 1 CNN (VGG16 [34]), good for 8 CNNs (ResNet50 [16], Densenet 121 [36], VGG16 + VGG19 + Reset152 + DenseNet169 [37], Inception-V3 [39], NasNet [41]), and excellent for 10 CNNs (EfficientNetB3 [35], ResNet50 + ResNet101 + DenseNet121 + DenseNet201 + InceptionV3 + EfficientNetB0 [37], ResNet152 [38], MobileNetV3 [2]).

Table 2 shows that while the same CNN architecture can have close results reported by different authors, they are not identical.

**Table 2:** Comparison of AUC results in identical CNN architectures according to different studies

	VGG 16	ResNet50	ResNet152	DenseNet121	Inception-V3
Tung et al [37]	0.86	0.91	0.88	0.93	0.93
Other author	0.77 [34]	0.84 [16]	0.92 [38]	0.87 [36]	0.88 [39]

Hendrix et al [39] assessed their algorithm’s performance using multiple input configurations for various X-ray projections including PA, ulnar-deviated PA, oblique, and lateral. The algorithm’s fracture detection performance improved when PA views were supplemented with ulnar-deviated PA views (AUC, 0.79 to 0.84), oblique views (AUC, 0.79 to 0.85), and all available views (AUC, 0.79 to 0.88). However, there was no significant improvement with the addition of lateral views.

**Fig. 5:**
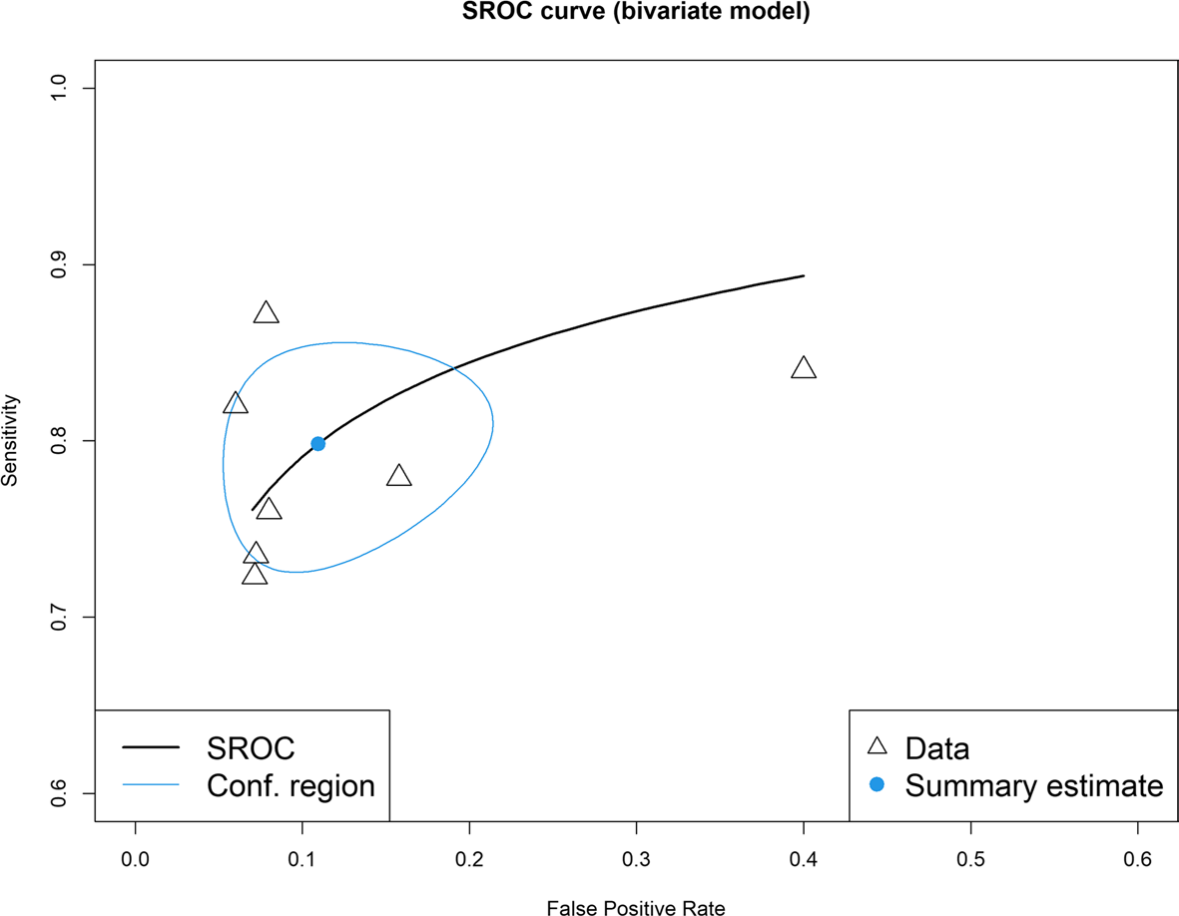
Summary Receiver Operating Characteristic (SROC) curve plot of deep learning models for the diagnosis of scaphoid fractures on wrist radiographs. Individual studies are shown as open triangles. Summary point is shown as an open circle, representing sensitivity estimates pooled by using bivariate random-effects regression model. The 95% confidence region (Conf. region) displays the 95% confidence interval of the pooled sensitivity and specificity. There was high heterogeneity among the studies, with *I*^2^ ranging from 80 to 90%

The Summary Receiver Operating Characteristic (SROC) curve plot of deep learning models for the diagnosis of scaphoid fractures on wrist radiographs is presented in Fig. 5. The area under the SROC curve is 0.88, pooled sensitivity is 80%, and specificity is 89%.

### Diagnosis of occult fractures

Langerhuizen et al [34] reported that 5 out of 6 occult scaphoid fractures missed by human observers were detected by AI. Contrary, Ozkaya et al [16] reported the same accuracy rate for AI and humans with similar 7 occult fractures which were missed by both. Yoon et al [35] developed two AI models, the first detecting both apparent and occult scaphoid fractures, and the second applied to negative-fracture images from the first model for occult fractures’ detection. The occult fracture model showed an AUC of 0.81 and the entire pipeline correctly identified 90.9% of the occult fractures.

Quality assessment using the QUADAS-2 tool is summarized in Table 3.

**Table 3:** Methodological analysis of the included studies based on the QUADAS-2 tool

Study	Risk of bias				Applicability concerns		
	Patient selection	Index test	Reference standard	Flow and timing	Patient selection	Index test	Reference standard
Langerhuizen et al [34]	High	Low	Low	Low	High	Low	Low
Ozkaya et al [16]	Unclear	Low	Low	Low	Unclear	Low	Low
Yoon et al [35]	High	Low	Unclear	Low	High	Low	Unclear
Hendrix et al [36]	High	Low	Low	Low	High	Low	Low
Tung et al [37]	Unclear	Low	High	Low	Unclear	Low	High
Yang et al [38]	Unclear	Low	High	Low	Unclear	Low	High
Li et al [2]	High	Low	Unclear	Low	High	Low	Unclear
Hendrix et al [39]	High	Low	Unclear	Unclear	High	Low	Unclear
Cohen et al [40]	High	Low	Unclear	Low	High	Low	Unclear
Lee et al [41]	High	Low	Unclear	High	High	Low	Unclear

## Discussion

The potential role of AI in the detection of radiographic scaphoid fractures is twofold. Firstly, it can help reduce missed scaphoid fractures, and secondly, it can aid in diagnosing occult scaphoid fractures. The current review and meta-analysis show that the overall diagnostic performance of AI in detecting scaphoid fractures is very good and near excellent. AI performance in this field demonstrates promising results in detecting both obvious and occult fractures. While studies comparing AI performance to human controls are lacking, existing data suggests that current AI systems are comparable to the performance level of human experts.

### AI features and diagnostic accuracy

The relatively limited number of studies evaluating the function of AI in the detection of scaphoid fracture on wrist radiographs and their recent publication (2020 or later) reflect the fact that this field is new, active, and evolving. CNN systems have evolved from the 1-step pipeline models, reliant on manual scaphoid segmentation, to 2-step pipeline models with integral CNN-based scaphoid segmentation [2, 16, 34–41].

The preliminary results show great promise: 10/19 and 8/19 CNNs show excellent and good AUC performance, respectively. Meaning, these models were able to accurately detect scaphoid fractures in plain radiography after training on considerably small datasets. The different scores of the CNNs may stem from different layer architecture and augmentation techniques. The difference between the same CNN in different studies might come from heterogenous input data, such as the number of images, data proportion in training/validation/test sub-sets, and image quality. The radiographic projections used in the AI systems also vary among studies, leading to diverse training and testing cohorts and different results.

In a recent review [42] that included 14 studies and evaluated the diagnostic performance of AI for detecting femoral neck fracture, a mean AUC of 0.969 was reported. The AUC score is somewhat higher compared to the current pooled SROC score. Yet, it is reasonable when taking into account the lower prevalence (2–10%) of occult proximal femur fractures [43] in comparison with scaphoid fractures.

### CNN performance compared to human performance

Results of the studies published so far have been promising, with AI systems showing a fair to excellent performance as measured in AUC [2, 16, 34–39, 41].

Only three studies [16, 34, 36] compared the AI diagnostic performance to human controls, in which, results varied. Langerhuizen et al [34] found no significant difference in the accuracy of diagnostic performance between the CNN and the performance of five orthopedic surgeons. Similarly, Hendrix et al [36] found no significant difference in AUC between the CNN and eleven radiologists. On the other hand, Ozkaya et al [16] found that an experienced orthopedic specialist performed better and had a higher AUC than the CNN.

Overall, the pooled sensitivity and specificity of the AI systems were 0.80 and 0.89, respectively. This suggests that AI systems have promising diagnostic performance. However, the human performance comparison groups and the information comprising the datasets in the various studies have a high degree of heterogeneity, making it difficult to draw clear conclusions on the relative performance of AI and human observers.

Three studies [39–41] conducted clinical validation studies, to compare human and AI diagnostic performance, and shed light on the potential benefits of utilizing AI in these contexts. Hendrix et al [39] found that while the AI system reduced reading time for certain radiologists, it did not lead to an improvement in the diagnostic performance for the majority of them. On the other hand, Cohen et al [40] reported that the implementation of the AI system increased sensitivity in fracture detection. Furthermore, Lee et al [41] demonstrated that the AI system significantly enhanced the diagnostic performance of two novice radiologists in identifying fractures. These studies collectively highlight the potential advantages of integrating AI technology in diagnostic processes, showcasing improvements in reading time and sensitivity for certain radiologists while also providing substantial enhancements in diagnostic capabilities for less experienced medical professionals.

The GRAD-CAM function was designed to integrate the AI output with current medical systems and allow clinicians to directly evaluate the AI output by highlighting the area of suspected fracture (heatmap). This function, other heatmaps, or any feature that delineates “zone-of-interest” may help direct clinical suspicion to an occult fracture, facilitating early diagnosis and treatment. Indeed, the GRAD-CAM function or similar features were utilized in five studies to highlight the area in the scaphoid most likely to be fractured, according to CNN [2, 35, 36, 40, 41]. The same function was previously utilized in AI systems developed to diagnose femoral neck fractures [42]. Cheng et al [44] reported that integrating this technology into the clinical flow is feasible and improves the diagnostic accuracy of physicians, especially novice clinicians. Sato et al [45] reported that using GRAD-CAM improved the accuracy, sensitivity, and specificity of resident-level physicians in the diagnosis of femoral hip fractures.

There are several limitations in the current review. The literature search was restricted to the English language. The available data sets for training CNNs are relatively small and heterogeneous and do not fully represent the complexity of real-life clinical cases. Furthermore, the CNN architectures differed between the AI systems, which also limits the ability to compare them. Additionally, none of the studies evaluated the performance of their algorithms in a clinical setting, where factors such as low-quality images or the presence of casts may affect diagnosis. Moreover, few studies compared the diagnostic performance of the AI systems to clinicians or radiologists; and only recent studies performed clinical validations to the AI systems.

The use of the QUADAS-2 tool in the included studies revealed a high risk of bias and concerns about applicability in 9 out of 10 studies.

These limitations highlight the need for further research and development in this field, including testing the algorithms in more diverse and representative image sets and evaluating their performance in real-life clinical settings.

In the current literature, there is a paucity of data regarding the use of AI in other modalities such as CT or MRI for detecting scaphoid fractures. This may be attributed to the fact that these modalities are typically deployed as confirmatory tools for suspected occult fractures, rather than being the initial or most commonly used modality for diagnosing and managing scaphoid fractures. Hence, the emphasis on AI development and research has been more pronounced in the context of X-ray imaging.

In conclusion, the current diagnostic performance of AI for detecting scaphoid fractures on wrist radiographs shows promising results, with a high pooled sensitivity and specificity and a high SROC result. AI systems cannot yet replace the human role in scaphoid fracture detection, but they may complement and augment the diagnostic performance of physicians. For novice healthcare practitioners, AI systems can improve the detection rate of fractures, especially in cases of occult fractures. For more experienced clinicians, AI systems may serve as a powerful diagnostic aiding tool, particularly when used in conjunction with heatmaps. Further research is required to establish a comparison between AI and human diagnostic performance in the clinical setting.

Still, existing AI systems can already be beneficial for non-expert clinicians in diagnosing both obvious and occult fracture; and aid experts in facilitating management when used in conjunction with heatmaps.

### Supplementary Information

Below is the link to the electronic supplementary material.

Supplementary file1 (PDF 281 KB)

## Abbreviations

AI: Artificial intelligence
AP: Anterior-posterior
AUC: Area under the ROC curve
CNN: Convolutional Neural Network
CT: Computed Tomography
GRAD-CAM: Gradient weighted Class Activation Map
IRR: Initial radiology reports
MRI: Magnetic resonance imaging
MSK: Musculoskeletal
PA: Posterior-anterior
PRISMA: Preferred Reporting Items for Systematic Reviews and Meta-analyses
QUADAS: Quality Assessment of Diagnostic Accuracy Studies
SROC: Summary Receiver Operating Characteristic

## Declarations

### Guarantor

The scientific guarantor of this publication is Dr. Eyal Klang.

### Statistics and biometry

One of the authors has significant statistical expertise.

### Study subjects or cohorts overlap

Some study subjects or cohorts have been previously reported in the studies that were included in the systematic review and meta-analysis.

### Methodology

• Retrospective

• Performed at one institution

## References

[1] Rhemrev SJ, Ootes D, Beeres FJ (2011). Current methods of diagnosis and treatment of scaphoid fractures. Int J Emerg Med.

[2] Li T, Yin Y, Yi Z, et al (2022) Evaluation of a convolutional neural network to identify scaphoid fractures on radiographs. J Hand Surg Eur. 17531934221127092. 10.1177/17531934221127092 10.1177/17531934221127092 36205038

[3] Steinmann SP, Adams JE (2006). Scaphoid fractures and nonunions: diagnosis and treatment. J Orthop Sci.

[4] Roolker W, Maas M, Broekhuizen AH (1999). Diagnosis and treatment of scaphoid fractures, can non-union be prevented?. Arch Orthop Trauma Surg.

[5] Prosser GH, Isbister ES (2003). The presentation of scaphoid non-union. Injury.

[6] Neviaser RJ (1986). On resection of the proximal carpal row. Clin Orthop Relat Res.

[7] Sabbagh MD, Morsy M, Moran SL (2019). Diagnosis and management of acute scaphoid fractures. Hand Clin.

[8] Shetty S, Sidharthan S, Jacob J, Ramesh B (2011). “Clinical scaphoid fracture”: is it time to abolish this phrase?. Ann R Coll Surg Engl.

[9] Balci A, Basara I, Çekdemir EY (2015). Wrist fractures: sensitivity of radiography, prevalence, and patterns in MDCT. Emerg Radiol.

[10] Welling RD, Jacobson JA, Jamadar DA (2008). MDCT and radiography of wrist fractures: radiographic sensitivity and fracture patterns. AJR Am J Roentgenol.

[11] de Zwart AD, Beeres FJP, Rhemrev SJ (2016). Comparison of MRI, CT and bone scintigraphy for suspected scaphoid fractures. Eur J Trauma Emerg Surg.

[12] Tiel-van Buul MM, van Beek EJ, Broekhuizen AH (1993). Radiography and scintigraphy of suspected scaphoid fracture. A long-term study in 160 patients. J Bone Joint Surg Br.

[13] Gibney B, Smith M, Moughty A (2019). Incorporating cone-beam CT into the diagnostic algorithm for suspected radiocarpal fractures: a new standard of care?. AJR Am J Roentgenol.

[14] Gäbler C, Kukla C, Breitenseher MJ (2001). Diagnosis of occult scaphoid fractures and other wrist injuries. Are repeated clinical examinations and plain radiographs still state of the art?. Langenbecks Arch Surg.

[15] Katzman BD, van der Pol CB, Soyer P, Patlas MN (2023). Artificial intelligence in emergency radiology: a review of applications and possibilities. Diagn Interv Imaging.

[16] Ozkaya E, Topal FE, Bulut T (2022). Evaluation of an artificial intelligence system for diagnosing scaphoid fracture on direct radiography. Eur J Trauma Emerg Surg.

[17] Wijetunga AR, Tsang VH, Giuffre B (2019). The utility of cross-sectional imaging in the management of suspected scaphoid fractures. J Med Radiat Sci.

[18] Klang E (2018). Deep learning and medical imaging. J Thorac Dis.

[19] Soffer S, Klang E, Shimon O (2020). Deep learning for wireless capsule endoscopy: a systematic review and meta-analysis. Gastrointest Endosc.

[20] Soffer S, Ben-Cohen A, Shimon O (2019). Convolutional neural networks for radiologic images: a radiologist’s guide. Radiology.

[21] LeCun Y, Bengio Y, Hinton G (2015). Deep learning. Nature.

[22] Litjens G, Kooi T, Bejnordi BE (2017). A survey on deep learning in medical image analysis. Med Image Anal.

[23] McBee MP, Awan OA, Colucci AT (2018). Deep learning in radiology. Acad Radiol.

[24] Klang E, Barash Y, Margalit RY (2020). Deep learning algorithms for automated detection of Crohn’s disease ulcers by video capsule endoscopy. Gastrointest Endosc.

[25] Barash Y, Klang E (2019). Automated quantitative assessment of oncological disease progression using deep learning. Ann Transl Med.

[26] Christopher M, Belghith A, Bowd C (2018). Performance of deep learning architectures and transfer learning for detecting glaucomatous optic neuropathy in fundus photographs. Sci Rep.

[27] Hosseinzadeh Kassani S, Hosseinzadeh Kassani P (2019). A comparative study of deep learning architectures on melanoma detection. Tissue Cell.

[28] Anteby R, Horesh N, Soffer S (2021). Deep learning visual analysis in laparoscopic surgery: a systematic review and diagnostic test accuracy meta-analysis. Surg Endosc.

[29] Anteby R, Klang E, Horesh N (2021). Deep learning for noninvasive liver fibrosis classification: a systematic review. Liver Int.

[30] Moher D, Liberati A, Tetzlaff J, Altman DG (2009). Preferred reporting items for systematic reviews and meta-analyses: the PRISMA statement. Ann Intern Med.

[31] McInnes MDF, Moher D, Thombs BD (2018). Preferred Reporting Items for a Systematic Review and Meta-analysis of Diagnostic Test Accuracy Studies: the PRISMA-DTA statement. JAMA.

[32] Whiting PF, Rutjes AWS, Westwood ME (2011). QUADAS-2: a revised tool for the quality assessment of diagnostic accuracy studies. Ann Intern Med.

[33] Reitsma JB, Glas AS, Rutjes AWS (2005). Bivariate analysis of sensitivity and specificity produces informative summary measures in diagnostic reviews. J Clin Epidemiol.

[34] Langerhuizen DWG, Bulstra AEJ, Janssen SJ (2020). Is deep learning on par with human observers for detection of radiographically visible and occult fractures of the scaphoid?. Clin Orthop Relat Res.

[35] Yoon AP, Lee Y-L, Kane RL (2021). Development and validation of a deep learning model using convolutional neural networks to identify scaphoid fractures in radiographs. JAMA Netw Open.

[36] Hendrix N, Scholten E, Vernhout B (2021). Development and validation of a convolutional neural network for automated detection of scaphoid fractures on conventional radiographs. Radiol Artif Intell.

[37] Tung Y-C, Su J-H, Liao Y-W (2021). High-performance scaphoid fracture recognition via effectiveness assessment of artificial neural networks. Appl Sci.

[38] Yang T-H, Horng M-H, Li R-S, Sun Y-N (2022). Scaphoid fracture detection by using convolutional neural network. Diagnostics (Basel).

[39] Hendrix N, Hendrix W, van Dijke K (2023). Musculoskeletal radiologist-level performance by using deep learning for detection of scaphoid fractures on conventional multi-view radiographs of hand and wrist. Eur Radiol.

[40] Cohen M, Puntonet J, Sanchez J (2023). Artificial intelligence vs. radiologist: accuracy of wrist fracture detection on radiographs. Eur Radiol.

[41] Lee K-C, Choi IC, Kang CH (2023). Clinical validation of an artificial intelligence model for detecting distal radius, ulnar styloid, and scaphoid fractures on conventional wrist radiographs. Diagnostics (Basel).

[42] Cha Y, Kim J-T, Park C-H (2022). Artificial intelligence and machine learning on diagnosis and classification of hip fracture: systematic review. J Orthop Surg Res.

[43] Deleanu B, Prejbeanu R, Tsiridis E (2015). Occult fractures of the proximal femur: imaging diagnosis and management of 82 cases in a regional trauma center. World J Emerg Surg.

[44] Cheng C-T, Ho T-Y, Lee T-Y (2019). Application of a deep learning algorithm for detection and visualization of hip fractures on plain pelvic radiographs. Eur Radiol.

[45] Sato Y, Takegami Y, Asamoto T (2021). Artificial intelligence improves the accuracy of residents in the diagnosis of hip fractures: a multicenter study. BMC Musculoskelet Disord.

