# Supplementary material for: Artificial intelligence for X-ray scaphoid fracture detection: a systematic review and diagnostic test accuracy meta-analysis
Source: Eur Radiol. 2023 Dec 15;34(7):4341–51. doi: 10.1007/s00330-023-10473-x (PMC11213739; doi:10.1007/s00330-023-10473-x)
Supplement: Supplementary file 1 — Supplementary file1 (PDF 281 KB) [file 330_2023_10473_MOESM1_ESM.pdf]

Table S1: Included studies methodological and numerical data

Study	Study Period	Imaging Modality	X-ray Exclusion Criteria	Number of CNN Pipeline Steps	CNN Sub-Purpose	Number of Patients	X ray views; per patient	Number of Images	Type of Fracture	Standard of Reference for Diagnosis	Human Comparison
**Langerhuizen et al [34]**	NA	X-Ray	Incomplete, distorted by cast or splint materials	1	Fracture Detection	300	4 (AP, PA, Lateral, Oblique Projections)	1200	Visible Fracture = 127<br>Occult Fracture = 23<br>No Fracture = 150	MRI-Confirmed or CT-Confirmed	5 Board-Certified Orthopedic Surgeons
**Ozkaya et al [16]**	NA	X-Ray	NA	1	Fracture Detection	390	1 (AP Projection)	390	Fracture = 192<br>No Fracture=198	Follow up CT scans	1 Emergency Doctor and 2 Orthopedic Hand Surgeons
**Yoon et al [35]**	January 2001 to December 2019	X-Ray	Poor quality, fractures older than 4 weeks, chronic hand conditions with bony changes around the scaphoid, psoriatic arthritis, rheumatoid arthritis, external immobilization (casts, splints, external fixations), hardware (screws, plates, wires, pins	3	Scaphoid Segmentation Model	4583	Exact number per patient not elaborated (PA or Scaphoid View)	11838	NA	A group of senior musculoskeletal radiologists provided final image interpretations. Radiographs with ambiguous or conflicting reports were reviewed by a hand surgeon. Final diagnoses were made based on the surgeon's interpretation. some cases had CT or MRI scans	NA
					Apparent Fracture Detection Model	4583		9533	Fracture = 3991<br>No Fracture = 5542		
					Occult Fracture Detection Model	547		695	Occult Fracture = 139<br>No Fracture = 556		
					Full Pipeline	NA		2305	Apparent Fracture = 904<br>Occult Fracture = 22<br>No Fracture = 1379		
**Hendrix et al [36]**	2017-2019	X-Ray	Old scaphoid fractures, screws or other implants, resection, excessive damage or malformation, cast	2	Scaphoid Segmentation	775	Exact number per patient not elaborated (AP or PA Projections of Hand, Wrist and Scaphoid Radiographs)	1039	NA	NA	NA
	2003-2019				Fracture Detection	1846		3000	Fracture = 1165<br>No Fracture = 1835	Original radiology reports. Dubious scaphoid fractures were reevaluated by a radiologist	NA
	2011-2020				Full Pipeline	190		190	Fracture = 95<br>No Fracture = 95	As Above and follow up CT scans	11 Radiologists, including: 3 residents, 6 Musculoskeletal (MSK) specialized senior Radiologists, 2 Non-MSK Senior radiologists
**Tung et al [37]**	NA	X-Ray	NA	2	Scaphoid Segmentation<br>Fracture Detection	154	Wrist Radiographs (Frontal or Lateral)	356	Fracture = 178<br>No Fracture = 178	Radiology Report	NA

**Yang et al [38]**	NA	X-Ray	NA	2	Scaphoid Segmentation	280	NA	361	Fracture = 167<br>No Fracture = 194	Surgical Verification	NA
					Fracture Detection	280		360	Fracture = 194<br>No Fracture =166		
**Li et al [2]**	2010-2020	X-Ray	Poor quality, screw or other implants, tumors affecting wrist bones, tuberculous or rheumatoid arthritis	2	Scaphoid Segmentation	500<br>Apparent Fracture -170<br>Occult Fracture – 60<br>No Fracture –270	Wrist Radiographs (PA or Scaphoid View)	930	Fracture = 513	For Occult Fractures: 61 had follow up CT Scans 11 had follow up MRI Scans	Majority decision of 4 hand surgeons In case of no majority, a 5th hand surgeon joined to obtain majority
					Fracture Detection	100<br>Apparent Fracture -38<br>Occult Fracture – 12<br>No Fracture – 50		209			
					Full Pipeline	600<br>Apparent Fracture -208<br>Occult Fracture - 72<br>No Fracture –320		1139	No Fracture = 626		
**Hendrix et al [39]**	2003-2019	X-Ray	Occlusion by casts, Occlusion by metallic osteosynthesis implants, Resection, not grown, unrecognizably deformed, non-acute fractures and severe scapholunate advanced collapse	2	Scaphoid Segmentation (Including laterality and x-ray projection detection)	394<br>Fracture -NA<br>No Fracture -NA	Hand, Wrist and Scaphoid Radiographs (AP/PA, Ulnar-deviated AP/PA, Oblique and Lateral)	1117	NA	2 MSK-specialized radiologists. Cases were independently reviewed. Disagreements were resolved by consensus reading. Some cases had follow up CT or MRI scans	5 experienced MSK radiologists with 5, 7, 22, 24 and 26 years of experience<br><br>Clinical Validation study: Interval between non-assisted radiograph reading and AI-assisted reading = 4 months
					Fracture Detection	209<br>Fracture -NA<br>No Fracture -NA		688 (different x-ray views) 219 Scaphoids (grouped by patients)	Fracture = 65<br>No Fracture = 154		

**Cohen et al [40]**	2017-2019	X-Ray	Braces or casts, technical limitations	Commercially available AI system ("BoneView" by Gleamer)	Scaphoid Segmentation & Fracture Detection are integral	637<br>Study tested for various wrist fractures, including scaphoid fractures	NA	1917	Scaphoid Fractures = 25	3 MSK-specialized radiologists with 5, 5 and 7 years of experience. Results were obtained by consensus.	Initial radiology results made by a total of 41 radiologists with various levels of experience, including:<br>29 residents (4th or 5th year of residency), 8 fellows in radiology, and 4 attendings.<br><br>Clinical Validation study<br>Comparison of diagnostic performance in 3 groups: AI, IRR & IRR+AI.<br>IRR+AI observation was considered positive when it was detected by either the AI or the IRR, regardless of the other's group result.
**Lee et al [41]**	2010-2020	X-Ray	Anatomic variation, bone abnormality, except for fractures (for example - tumor), splint or cast, orthopedic hardware, poor image quality	2 Parallel CNNs integrated to final result	Scaphoid Segmentation<br><br>Fracture Detection	593<br>Study tested for various wrist fractures, including scaphoid fractures	Exact number per patient not elaborated.<br>Wrist Radiographs (AP, Lateral, Oblique)	1186	Scaphoid Fractures = 32	1 MSK-specialized radiologist and 1 orthopedic upper-limb surgeon; both with 19 years of experience.	2 radiologists:<br>2-year-fellowship-trained musculoskeletal radiologist<br>1-year-trained radiology resident<br><br>Clinical Validation study<br>Interval between non-assisted radiograph reading and AI-assisted reading = 3 weeks

NA = Not Available
MSK =Musculoskeletal
AI = Artificial Intelligence
IRR = Initial Radiology Reports

Table S2: Included Studies artificial intelligence characteristics

Study	Fracture Detection - CNN Architecture Type	Scaphoid Segmentation	ROI or important region labeling	Data Input proportion in training / validation / test	Augments
**Langerhuizen et al [34]**	VGG16	Rectangular ROI wrapping the scaphoid. Manually cropped and resized.	NA	Training - 180<br>Validation - 20<br>Test - 100	10-fold augmentation of the training and validation set by using rotation (-15° and +15°), shifting of height and width (10%), zooming (between 0.8 and 1.1), and horizon flipping. The test set only composed of original radiographs.
**Ozkaya et al [16]**	ResNet50*	Rectangular ROI wrapping the scaphoid. Marked by Expert radiologist	NA	Training - 203<br>Validation - 87<br>Test - 100	NA
**Yoon et al [35]**	EfficientNetB3	Scaphoid detection model based on Cascade Region-based Convolutional Neural Network; was trained and used to isolate the scaphoid in a bounding box in hand radiographs	Highlight important regions = Grad-CAM	Total Pipeline<br>Training - 8356<br>Validation - 1177<br>Test - 2305<br>Apparent Fracture Model<br>Training - 8306<br>Validation - 1151<br>Occult Fracture Model<br>Training - 565<br>Validation - 130	random flip, scale, random brightness, rotation (<15°), flip, resizing, and standardization.
**Hendrix et al [36]**	DenseNet-121	"Segmentation CNN"	Highlight important regions = Grad-CAM	NA	Horizontal Flip, Image Shift, Bounding Box Shift, Scale, Rotate, Grid Distortion, Elastic Transform, Brightness, Contrast
**Tung et al [37]**	VGG16, VGG19, ResNet50, ResNet101, ResNet152, DenseNet121, DenseNet169, DenseNet201, Inception-V3, EfficientNetB0	YOLOv4	NA	Training + Validation - 70%<br><br>Test - 30%	Flip, Rotaion +/- 15
**Yang et al [38]**	ResNet-152	RCNN +ResNet50<br>Cascade R-CNN (Region-based Convolutional Neural Network)<br>marked the fracture in a bounding box	NA	NA	Contrast limited adaptive histogram equalization (CLAHE),<br>Random horizontal flip with 50% probability,<br>Random contrast with 50% probability
**Li et al [2]**	MobileNetV3	YOLOv3	Highlight important regions = Grad-CAM	Training + Validation - 930<br>Test -209	NA
**Hendrix et al [39]**	Inception-V3	YOLOv5s	NA	Scaphoid localizer and laterality classifier:<br>Training + Validation – 12990<br>9% overlap with prior work by Hendrix et al [36]<br>Scaphoid fracture detector:<br>Training + Validation – 4316<br>82% overlap with prior work by Hendrix et al [36]<br>Test – 688<br>5% overlap with prior work by Hendrix et al [36]	Horizontal flipping<br>Rotation<br>Gaussian Noise

**Cohen et al [40]**	Detectron 2 Framework	NA	Zone of interest, integral to the AI system:<br>"solid line box" considered as a certain fracture with a high level of specificity<br>"dotted line box" considered as a probable fracture with a high level of sensitivity	"BoneView" by Gleamer<br>(commercially available AI system)<br>60170 radiographs:<br>Training - 70%<br>Validation - 10%<br>Internal tests - 20%	NA
**Lee et al [41]**	NasNet	RetinaNet	Heat Map	MURA dataset (public radiographic dataset) – 3791 for distal radius & ulnar styloid fractures<br><br>Hospital dataset – 641 for scaphoid fractures<br>validation set - 20%	Contrast limited adaptive histogram equalization (CLAHE)<br>Normalization<br>Histogram Matching<br>Sharpening

* Replaced the uppermost layers with a set of layers that performed two class classifications.

NA = Not Available
AI = Artificial Intelligence

Table S3: Included Studies Diagnostic Test Accuracy, and comparison to human performance

Study	AI Fracture Diagnosis					Human Fracture Diagnosis				
	CNN	Accuracy	AUC	Sensitivity	Specificity	Experience	Accuracy	AUC	Sensitivity	Specificity
**Langerhuizen et al [34]**	VGG16	72.00%	0.77	84.0%	60.0%	Board-Certified Orthopedic Surgeons	84.0%	NA	76.0%	93.0%
**Ozkaya et al [16]**	ResNet50*	NA	0.84	76.0%	92.0%	Experienced Orthopedic Surgeon	NA	0.92	86.0%	98.0%
						Less Experienced Orthopedic Surgeon	NA	0.82	72.0%	92.0%
						Emergency Doctor	NA	0.76	62.0%	90.0%
**Yoon et al [35]**	EfficientNetB3	NA	Total Pipeline			NA				
			X	97.2%	66.0%					
			Apparent Fracture Model							
			0.955	87.1%	92.1%					
			Occult Fracture Model							
			0.81	79.0%	71.6%					
**Hendrix et al [36]**	DenseNet-121	NA	Fracture Detection			NA				
			0.86	66.0%	90.0%					
			Entire System Test			Average of 11 Radiologists [Range]	NA	0.83 [0.79-0.87]	NA [54.0-80.0%]	NA [75.0-95.0%]
			0.87	78.0%	84.0%					
**Tung et al [37]**		Fracture Detection				NA				
	VGG 16	83.3%	0.86	86.1%	80.6%					
	VGG19	77.8%	0.87	83.3%	72.2%					
	ResNet50	86.1%	0.91	88.9%	83.3%					
	ResNet101	88.9%	0.95	88.9%	88.9%					
	ResNet152	80.6%	0.88	80.6%	80.6%					
	DenseNet121	87.5%	0.93	91.7%	83.3%					
	DenseNet169	88.9%	0.89	91.7%	86.1%					
	DenseNet201	90.3%	0.91	94.4%	86.1%					
	Inception-V3	88.9%	0.93	88.9%	88.9%					
	EfficientNetB0	86.1%	0.92	94.4%	77.8%					
**Yang et al [38]**	ResNet-152	Detection of Fracture Area				NA				
		85.3%	0.92	78.9%	90.0%					
		Fracture Detection								
		82.9%	0.917	73.5%	92.0%					
**Li et al [2]**	MobileNetV3	NA	0.919	82.0%	94.0%	Majority between hand surgeons	NA	NA	76.0%	96.0%

**Hendrix et al [39]**	Inception-V3	NA	All available X-ray projections			Average of 5 MSK Radiologists [Range]	NA	0.87	[75.0%-83.0%]	[81.0%-94.0%]
			0.88	72.0%	93.0%					
			PA							
			0.79	51.0%	93.0%					
			PA + ulnar-deviated PA							
			0.84	59.0%	93.0%					
			PA + oblique							
			0.85	66.0%	93.0%					
			PA + lateral							
			0.83	55.0%	93.0%					
**Cohen et al [40]**	"Bone View" by Gleamer	NA	NA	For scaphoid subset = 84.0%	NA	Initial Radiology Reports	NA	NA	80.0%	NA
**Lee et al [41]**	NasNet	74.0%	0.81	87.0%	74.0%	2-year-fellowship-trained MSK radiologist without AI assistance	98.0%	0.75	50.0%	99.0%
						2-year-fellowship-trained MSK radiologist with AI assistance	98.0%	0.85	72.0%	98.0%
						1-year-trained radiology resident without AI assistance	94.0%	0.71	47.0%	96.0%
						1-year-trained radiology resident with AI assistance	93.0%	0.80	66.0%	94.0%

* Replaced the uppermost layers with a set of layers that performed two class classifications.
NA = Not Available
MSK = Musculoskeletal
AI = Artificial Intelligence

Table S4: Key for stratification and grading of the included studies using the QUADAS-2 tool

**Patient Selection**	**High**	**Exclusion of "difficult to diagnose" cases, for example radiographs with casts.**
	**Unclear**	**No specification of exclusion criteria.**
	**Low**	**Full random sampling of eligible patients /radiographs.**
**Index Test**	**Low**	**The AI systems are not aware of the "ground truth" results, so they were all deemed low.**
**Reference Standard**	**High**	**Not stated clearly if fractures and non-fractures were confirmed by either CT or MRI, or by expert/s.**
	**Unclear**	**Fractures and non-fractures were assessed by expert/s. Some cases or none, were confirmed by CT or MRI.**
	**Low**	**Fractures and non-fractures were confirmed by either CT or MRI.**
**Flow and Timing**	**High**	**Clinical validation studies with a relatively short period of time (less than a month) between non-AI assisted readings, compared with AI-assisted readings.**
	**Unclear**	**Clinical validation studies with over a month time between non-AI assisted readings, compared with AI-assisted readings.**
	**Low**	**Studies without clinical validation, in which there is no need for an appropriate interval in between consecutive readings of radiographs.**
